# The bacterial proteogenomic pipeline

**DOI:** 10.1186/1471-2164-15-S9-S19

**Published:** 2014-12-08

**Authors:** Julian Uszkoreit, Nicole Plohnke, Sascha Rexroth, Katrin Marcus, Martin Eisenacher

**Affiliations:** 1 Medizinisches Proteom-Center, Ruhr-Universität Bochum, Bochum, Germany; 2 Plant Biochemistry, Ruhr-Universität Bochum, Bochum, Germany

**Keywords:** proteogenomics, proteomics, mass spectrometry, peptide identification, genome annotation

## Abstract

**Background:**

Proteogenomics combines the cutting-edge methods from genomics and proteomics. While it has become cheap to sequence whole genomes, the correct annotation of protein coding regions in the genome is still tedious and error prone. Mass spectrometry on the other hand relies on good characterizations of proteins derived from the genome, but can also be used to help improving the annotation of genomes or find species specific peptides. Additionally, proteomics is widely used to find evidence for differential expression of proteins under different conditions, e.g. growth conditions for bacteria. The concept of proteogenomics is not altogether new, in-house scripts are used by different labs and some special tools for eukaryotic and human analyses are available.

**Results:**

The Bacterial Proteogenomic Pipeline, which is completely written in Java, alleviates the conducting of proteogenomic analyses of bacteria. From a given genome sequence, a naïve six frame translation is performed and, if desired, a decoy database generated. This database is used to identify MS/MS spectra by common peptide identification algorithms. After combination of the search results and optional flagging for different experimental conditions, the results can be browsed and further inspected. In particular, for each peptide the number of identifications for each condition and the positions in the corresponding protein sequences are shown. Intermediate and final results can be exported into GFF3 format for visualization in common genome browsers.

**Conclusions:**

To facilitate proteogenomics analyses the Bacterial Proteogenomic Pipeline is a set of comprehensive tools running on common desktop computers, written in Java and thus platform independent. The pipeline allows integrating peptide identifications from various algorithms and emphasizes the visualization of spectral counts from different experimental conditions.

## Background

High throughput bottom-up proteomics using LC-MS [1] has become one of the major proteomics approaches today. In this technique tandem MS (MS/MS) spectra are usually matched by search or identification algorithms to peptide sequences in protein databases. The databases used contain protein sequences with varying quality: only a minor part of the sequences are experimentally validated, some are predicted, e.g. by homology to other species, while a considerable part of the sequences are only based on predicted open reading frames. Protein prediction algorithms are very advanced, but still have weaknesses for the prediction of small proteins, introns and translation start sites. For most exotic species not commonly used in the lab, there are no well curated protein databases at all.

As bacterial genomes are comparatively short and thus cheap to sequence, it is feasible to create protein databases by translating all six reading frames of the genome. We call the proteins originating from this direct translation "pseudo proteins" in this work, whereas annotated proteins are referred to as "known proteins". Such a database containing pseudo and known proteins can be used to identify MS/MS spectra, which cannot be identified in conventional databases or deriving from species without protein databases. This approach is called proteogenomics [2,3] and allows enhancing the annotation of the genome of the analyzed species as well as the improvement of existing protein databases. These enhancements may include the correction of predicted reading frame boundaries as well as the discovery of new proteins or peptides.

There are already several approaches for proteogenomic tools: some try to tackle the very large number of pseudo proteins generated from eukaryotic genomes [3-5], others developed new, specialized search engines for this task, as shown in [6] and [7]. Almost all tools, including e.g. the GenoSuite [8], allow only a small set of search algorithms for peptide identification. To the best of our knowledge, there is no standalone tool which allows the visualization and comparison of pseudo peptides found in different experimental conditions and which imports identifications from mzTab [9] format and thus supports any peptide identification, combination of identification algorithms or post-processing algorithm. For further inspection of the results and all intermediate information, all protein and peptide information can be exported to the Generic Feature Format 3 (GFF3), which is widely supported by common genome browsers.

## Implementation

**Figure 1:**
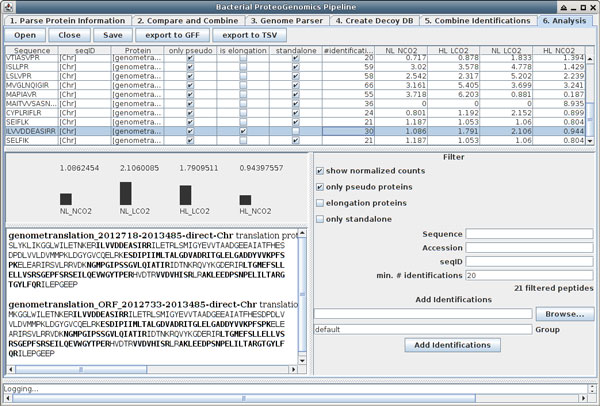
**Screenshot of the Bacterial Proteogenomic Pipeline GUI**. The GUI of the Bacterial Proteogenomic Pipeline leads the user through all steps required for a proteogenomic analysis. Shown is the final step, the analysis of the combined search results. After opening a file created in the "Combine Identifications" step, the identified peptide sequences are shown in a table with information about the sequence, the originating genomic sequence (usually the chromosome or a plasmid), corresponding protein accessions, whether or not the peptide occurs only in a pseudo protein, in an elongation of an annotated protein or is a standalone pseudo protein. Additionally the numbers of distinct identifications in all files and the (normalized) numbers of identifications per condition of the searched samples are given and represented in the bar charts in the lower half of the screen. For a selected peptide, the protein sequences containing the peptide are depicted, with the identified sequences highlighted in bold. The result table can be filtered and additional spectrum identification files can be added, for which the condition groups may be freely chosen.

The Bacterial Proteogenomic Pipeline consists of several Java classes which allow a complete proteogenomics approach using MS/MS data, except for the peptide identification step, which is done by search engines. All parts of the pipeline can be run on any current desktop system compatible with Java. The source code is available under a three-clause BSD license and thus open source for everyone. Besides the command line execution, we provide a GUI which will guide the user in six steps through the analysis. The steps will be further explained in the following paragraphs. Figure 1 shows the GUI at the last analysis step (i.e. the listing and visualization of the identified peptides).

### Step 1: Parse protein information

In this first step, the protein information of the already annotated known proteins respectively their genes is parsed either from a separated values file (commonly a tab, TSV, or comma, CSV, separated values file) or a protein FASTA file and saved into a GFF3 file. For each gene or protein the accession, the genomic start and end positions and the strand information (forward or reverse) must be included in the file and will be parsed. Additionally a protein/gene description and the originating chromosome or plasmid name may be obtained. For a TSV file, only the column for each parsed variable must be defined. For a FASTA file, regular expressions of how to get the information from the gene or protein header are used. For the pipeline to be able to gather all information correctly, the FASTA file, which contains the known proteins, should have the same accessions as the accessions parsed in this step.

### Step 2: Compare and combine

This optional step allows adding further protein information from a reference FASTA file, additionally to the one containing the known proteins' information generated in step 1. This is for example interesting, if the FASTA file for the known proteins originates from a species specialized database and the accessions and sequence information from e.g. the UniProt KB should be added to the known proteins. Also the proteins of a host species (for e.g. symbiotic or pathogenetic species) or a contaminant database can thus be merged to the list of known proteins.

There are two ways to find related entries in the protein list parsed in step 1 and an additional reference FASTA: either a given mapping file between the accessions of the lists may be used or, if for an entry no mapping is found, the amino acid sequences are compared. In the latter case a relation between the proteins is assumed only if the difference between the lengths of the sequences is not bigger than 100 amino acids. Three kinds of relations are identified and added to the description of the protein: "equal to X" if the protein sequences are identical, "elongation by X" if the reference protein has a longer amino acid sequence (but completely contains the target protein's sequence) and "elongation of X" if the reference protein's sequence is shorter and contained in the target protein's sequence (X represents the respective reference protein's accession). If an "elongation" relation is detected, the longer sequence is stored. For any protein, which cannot be related or mapped to a known protein, the information from the reference file is copied. The combination finally creates new FASTA and GFF3 files for the subsequent steps.

### Step 3: Genome parser

The Genome Parser creates the naïve six frame translated protein FASTA database of a given genome. The translation starts on the first position of the genome and reads nucleotide triplets until the first stop codon is reached. Immediately after the stop codon is reached, a new pseudo protein is started instead of waiting for the next start codon to appear. If at least one start codon exists (open reading frame, ORF) in the pseudo protein, additionally the longest ORF will be translated and written to the FASTA file (these proteins are called "ORF pseudo proteins"). It is necessary to also have these ORF pseudo proteins starting with a methionine translated from the start codon to allow the search engine to correctly match possible MS/MS spectra against the respective N-terminal peptides. Unfortunately, this approach creates a set of overlapping proteins for each start codon which does not immediately follow a stop codon and thus increase the time needed for the spectrum identification. The Bacterial Proteogenomic Pipeline uses the codons ATG, TTG, CTG, ATT, ATC, ATA and GTG as start codons, which in the case of a start codon are all translated into methionine. If the positions of the known proteins are given, proteins translating from exactly the same genome site will not be added to the pseudo proteins to avoid redundancy. Pseudo proteins overlapping one or more annotated proteins are tagged appropriately in their description with "elongation of", similar as described in step 2.

### Step 4: Create decoy database

This step is optional and assists the user in building a decoy database containing shuffled decoy entries of the target entries to perform target-decoy searches [10,11]. Either a concatenation of target and decoy entries or a single database with decoy entries only can be created.

After the search database respectively databases (if the chromosome and several plasmids of one species were translated) containing both known and pseudo proteins are created, the peptide identification of the MS/MS data can be performed by any search algorithm, e.g. SEQUEST [12], MS-GF+[13], Mascot [14] or X!Tandem [15]. This must be performed by the user manually and thus also gives free choice of any validation and filtering using certain FDR or other criteria. After the identification and validation/filtering, the identified peptide spectrum matches passing the criterions must be exported into mzTab files, one for each MS/MS run. For the export e.g. OpenMS [16] or PIA (https://github.com/mpc-bioinformatics/pia) can be used, which are both open software.

### Step 5: Combine identifications

In this step the results of the peptide identifications can be grouped into sets representing any kind of experimental condition, like e.g. different growth conditions of the samples. The identifications are parsed from mzTab files, combined and can be saved into a SQLite database for subsequent analysis. Additionally, the combined data can directly be written into two GFF3 files, one containing only the peptides of pseudo proteins, the other all remaining peptides. A peptide is defined by the amino acid sequence only, neglecting any modifications or charge states. For each peptide in the GFF3 file there will be one feature for each condition group with the score set to the respective number of identified spectra and one feature for the overall number of identifications.

### Step 6: Analysis

The final step, which is only available in the GUI and depicted in Figure 1, is for a manual review and analysis of the results. For each peptide, the corresponding proteins are shown and whether they are originating from the genome or plasmids. Furthermore it is stated whether the peptide was found in pseudo proteins only and whether these proteins are an elongation of any known protein or are standalone pseudo proteins, i.e. proteins without any overlap to a known protein. The number of identified different spectra for each peptide, also called spectral counts, is given as sum of all imported files and additionally for each assigned group. For the assigned groups, the counts can also be shown normalized. This normalization makes the assumption that the total amount of identifiable protein is equal per sample and is performed by the following operation

$${c^{\prime }}_{i}={\text{max}}_{t}\left(n_{ft}\right)\times \frac{c_{i}}{n_{fi}},$$

where $c_{i}$ is the raw count for peptide  i and $n_{fi}$ is the total number of counts in the respective identification file. To obtain human readable values, the quotient is multiplied by the largest number of counts of all individual files ($\underset{t}{\max}\left(n_{ft}\right)$). For a better perception, the distribution of counts per group is also visualized in a bar chart. If the full sequence of a protein is known, it is visualized with the sequences of the identified peptides highlighted to help in assessing the relevance of identifications. The analysis allows several filters to show e.g. identifications of pseudo proteins only or only peptides, which exceed a given number of identifications. The Bacterial Proteogenomic Pipeline also allows adding of further identification files in this step to enhance an analysis and the export into GFF3 files as discussed in the previous paragraph or a simple tab separated format.

## Results and discussion

The Bacterial Proteogenomics Pipeline was tested on two datasets, one publicly available containing data from *Bradyrhizobium Japonicum *(an endosymbiont of legumes) samples grown in cowpea nodules ([17], PRIDE accessions 10099-10101) and one containing *Synechocystis sp. PCC 6803 *(hereafter: *Synechocystis*) samples, which were cultivated under different environmental conditions.

For the *B. Japonicum *dataset, the genome and known proteins were downloaded from the NCBI using the reference sequence NC_004463.1 (8317 protein entries, downloaded on 14.05.2014) and processed by the Bacterial Proteogenomic Pipeline. The proteins of *Vigna unguiculata *(cowpea), which acted as host, were downloaded from the UniProt (release 2014_5, 379 entries) and added to the list of known proteins, as well as "The common Repository of Adventitious Proteins, cRAP" (115 entries, unchanged since 29.02.2012), resulting in a total of 8811 known proteins. The Genome Parser created 505804 pseudo proteins. From these databases (all together 514615 entries), a target decoy database was created and searched by X!Tandem and MS-GF+, using fixed carbamidomethylation of cysteine and variable oxidation of methionine as modifications. The results were combined with PIA (https://github.com/mpc-bioinformatics/pia) and only PSMs with Combined FDR Scores [18] below 0.01 were exported to mzTab files. The three resulting mzTab files were further processed by our software and for the analysis the minimal number of identifications per peptide was set to 5. With these rather strict settings, we detected all together 32 new peptides, of which 4 represent protein elongations respectively gene boundary changes and 28 completely new proteins, all peptides are proteotypic (i.e. identified only in one protein, though some in an ORF pseudo protein and the respective pseudo protein as well) given the databases used. Most but not all of these new identifications were also found by Kumar et al. in [8], the list of peptides is shown in table 1. All necessary steps except for the spectrum identification were carried out using the GUI on a laptop computer (Intel i7 M620 CPU running on 2.66 GHz, 8 GB RAM of which Java was allowed to use 2 GB) in a few minutes (step1: <1 s, step 2: ~4 s, step 3: ~28 s, step 4: ~8 s, step 5: ~55 s, opening file for analysis: ~24 s). The time needed for the spectrum identification depends on the used search engine(s) and data and therefore cannot be estimated accurately in general, but for this test sample and the prior stated search parameters took about two hours.

**Table 1:** Peptides found in the *B. Japonicum* analysis

Sequence	number of identifications (normalized)	elongation / standalone	ORF start	ORF end	**reported in **[8]
VLVEGIER	5 (2.62)	standalone	**498334**	**498939**	

FSDYAFPPAVGYPSFAR	23 (14.78)	standalone	*539034*	*539441*	yes

GRPVYGPSGPNTVYQQGR	15 (10.79)	standalone	*539034*	*539441*	yes

KADLEAR	24 (12.65)	standalone	**1313439**	**1314140**	

ALVAEISR	6 (3.02)	standalone	*1863514*	*1863603*	

APPIEPR	7 (5.19)	elongation	**1926621**	**1927364**	

ASVQYFVTR	7 (5.40)	standalone	*2056995*	*2057228*	yes

VAVDAAHK	6 (3.41)	standalone	*2056995*	*2057228*	yes

VAVDAAHKEGK	5 (3.01)	standalone	*2056995*	*2057228*	yes

IGELAEATGVTVR	9 (6.21)	elongation	**2179134**	**2179862**	

ALNLGIGLGHQR	10 (7.00)	standalone	*2241275*	*2241463*	yes

VIESDAGDGER	6 (4.99)	standalone	**2320354**	**2320803**	yes

ASADPAPSPAEAER	5 (3.40)	standalone	**2320354**	**2320803**	yes

LAASQCPVAAIR	5 (3.01)	standalone	**2320354**	**2320803**	yes

TTMEQATAAAK	14 (7.63)	standalone	*2672562*	*2672918*	yes

LQMSADNVADSYAR	6 (3.80)	standalone	*2672562*	*2672918*	yes

ADADLDVVIR	5 (3.40)	standalone	*2672562*	*2672918*	yes

MVDCRIK	5 (2.41)	standalone	**3263474**	**3263848**	

AAEGTLR	6 (4.01)	standalone	*3686105*	*3687250*	

VIAGEQGAQR	5 (3.40)	standalone	**4603312**	**4603641**	yes

ILVLYGSYR	5 (3.60)	standalone	*4634660*	*4635250*	yes

VLDASTAYR	5 (3.99)	standalone	**4817856**	**4819223**	yes

CYQSAAAYVGQDR	7 (4.21)	standalone	*5865762*	*5866031*	yes

LVQIQCER	5 (2.62)	standalone	**6019469**	**6026782**	

GNALLNFGK	5 (3.40)	standalone	*6030625*	*6031395*	

AGSTPIPSAEAPDR	5 (3.40)	standalone	**6676399**	**6676560**	yes

GQGEGAPGQASDR	9 (4.42)	elongation	*7177670*	*7178182*	

VVSKPLPTFTAASDLQIK	16 (11.60)	standalone	**7341856**	**7342332**	yes

YKPFQWGASTYK	5 (2.80)	standalone	**7341856**	**7342332**	yes

LILAEPAPGVR	5 (3.60)	standalone	*8111257*	*8112012*	yes

AVGVLAAEYLR	6 (4.40)	elongation	**8250513**	**8251328**	

GCITPQTGRGQAASPVR	16 (9.03)	standalone	*8914192*	*8914341*	

This table shows the peptides of pseudo proteins found in a proteogenomic analysis of *B. Japonicum*. MS/MS spectra were identified with MS-GF+ and X!Tandem, the combined search results were filtered on a Combined FDR Score level of 0.01. Only peptides, which had at least 5 distinct peptide spectrum matches are reported, peptides from the same ORF respectively pseudo protein are visually grouped by the alternating bold and recursive ORF positions.

The analysed *Synechocystis *cultures were grown under four different environmental conditions: normal (NL) and high light (HL) each combined with normal (NCO2) and high CO2 (HCO2) levels. The genome and protein information was downloaded from the Cyanobase (http://genome.microbedb.jp/cyanobase/Synechocystis) together with sequences for the plasmids pSYSA, pSYSG, pSYSM, pSYSX, sequences for pCA2.4, pCB2.4 and pCC5.2 were downloaded from the NCBI sites. This information was enriched by protein information from the UniProt by the "Compare and Combine" module (step 2 of the analysis). Eight samples of each condition were measured and the resulting MS/MS spectra matched against a target-decoy database of the known and pseudo proteins with Mascot, MS-GF+ and X!Tandem. The results were combined and filtered as described in the previous paragraph. A thorough analysis of the (differentially expressed) identified pseudo proteins is pending. The Bacterial Proteogenomic Pipeline detected 47 peptides found in pseudo proteins with at least 10 distinctive identified spectra, of which 2 elongate known proteins and 45 belong to new standalone proteins, 4 of these peptides are not proteotypic, but could be associated to more than one pseudo protein.

Besides the further analysis of the *Synechocystis *dataset, further improvements of the Bacterial Proteogenomic Pipeline may include the visualization of annotated spectra and the direct import of more standard formats like mzIdentML and filtering of used identifications inside the pipeline.

## Conclusions

We presented the Bacterial Proteogenomic Pipeline, a set of tools for proteogenomics analyses with emphasize on the visualization of results, which runs on current desktop computers and allows an operating system independent execution. The usage of a standard format for the spectrum identifications import allows the user to run virtually any peptide identification and post processing algorithm. The results of a processed analysis can be browsed via the provided GUI or can be exported into GFF3 files and imported into any common genome browser.

## Availability and requirements

**Project name**: Bacterial Proteogenomic Pipeline

**Project homepage**: https://github.com/mpc-bioinformatics/bacterial-proteogenomic-pipeline

**Operating system(s)**: Platform independent (Java)

**Programming language**: Java

**Other Requirements**: Java 1.5

**License**: Three-clause BSD license

**Any restrictions to use by non-academics**: None

## Competing interests

The authors declare that they have no competing interests.

## Authors' contributions

JU did the implementation of the GUI and drafted the manuscript, JU and ME programmed together all other implementations. ME and SR had the initial idea for the Bacterial Proteogenomic Pipeline. SR additionally provided the samples for the Synechocystis, which were cultivated, processed and measured on the mass spectrometers by NP. KM provided the computational infrastructure and contributed to the background and discussion parts.

## List of abbreviations

FDR: False Detection Rate

GFF3: Generic Feature Format 3

ORF: open reading frame

## Declarations

Funding for the publication of this article was provided by PURE (Protein Unit for Research in Europe), a project of North Rhine-Westphalia, Germany.

This article has been published as part of *BMC Genomics *Volume 15 Supplement 9, 2014: Thirteenth International Conference on Bioinformatics (InCoB2014): Computational Biology. The full contents of the supplement are available online at http://www.biomedcentral.com/bmcgenomics/supplements/15/S9.
